# Microvascular Invasion in HCC: The Molecular Imaging Perspective

**DOI:** 10.1155/2018/9487938

**Published:** 2018-10-04

**Authors:** Vincenzo Cuccurullo, Giuseppe Danilo Di Stasio, Giuseppe Mazzarella, Giuseppe Lucio Cascini

**Affiliations:** ^1^Nuclear Medicine Unit, Department of Precision Medicine, Università della Campania “Luigi Vanvitelli”, Napoli, Italy; ^2^Department of Diagnostic Imaging, Nuclear Medicine Unit, Magna Graecia University of Catanzaro, Catanzaro, Italy

## Abstract

Hepatocellular carcinoma represents the most frequent primary liver tumor; curative options are only surgical resection and liver transplantation. From 1996, Milan Criteria are applied in consideration of patients with cirrhosis and hepatocellular for liver transplantation; nonetheless, more recently, Milan Criteria have been criticized because they appear over conservative. Apart from number and size of lesions and biomarker levels, which already have been associated with poorer prognosis, overall survival and recurrence rates after transplantation are affected also by the presence of vascular invasion. Microvascular invasion suggests a poor prognosis but it is often hard to detect before transplant. Diagnostic imaging and tumor markers may play an important role and become the main tools to define microvascular invasion. In particular, a possible role could be found for computed tomography, magnetic resonance imaging, and positron emission tomography. In this paper, we analyze the possible role of positron emission tomography as a preoperative imaging biomarker capable of predicting microvascular invasion in patients with hepatocellular carcinoma and thus selecting optimal candidates for liver transplantation.

## 1. Introduction

Hepatocellular carcinoma (HCC) represents the most frequent primary liver tumor being about 90% of all cases of liver cancer [1]. In recent years, we witnessed an increase in its prevalence rates up to today, when it is the fifth most common cancer and the second leading cause of cancer-related deaths worldwide [2]. The curative options for this type of cancer are surgical resection and liver transplantation (LT) [3]; however, the former is not always feasible due to tumor-related cirrhosis, which limits major hepatectomy, and the latter is hampered by shortage of organs and HCC recurrence after LT, which happens in up to 75% of patients [4]. In order to overcome these problems and improve patient selection, Mazzaferro et al. published in 1996 the first version of the so-called Milan criteria (MC) [5], which demonstrated in 48 patients that selecting cases for transplantation according to specific strict criteria led to an improvement in both overall and disease-free survivals. These criteria take in account the morphological parameters such as size and number of nodules (one lesion smaller than 5 cm or, alternatively, up to three lesions, each smaller than 3 cm), with diagnostic imaging techniques like contrast CT and MRI that act as main tools used to reach diagnosis and prognostic evaluation of HCC [6]. Nonetheless, more recently, MC have been criticized because they appear over conservative, thus refraining a significant number of patients from a possible curative treatment [7]. In this sense, nowadays, it is generally accepted that MC cutoffs have to be extended in order to increase the number of HCC patients eligible for LT [8]. However, it is still unclear how far the selection limits may be pushed without excessively increasing the risk of tumor relapse. Currently, a minimum survival probability between 50% and 60% at 5 years after LT is demanded in order to balance benefit and harm of LT beyond standard criteria, with microvascular invasion (MVI) [9, 10] and low tumor differentiation that are recognized as most important prognostic factors, together with serum levels of alphafetoprotein (AFP) and response to neoadjuvant locoregional tumor treatment (LRTT) [11, 12]. In particular, Lim et al. reported that MVI is an independent predictor of tumor recurrence after surgical resection, more prominent than MC, with its presence being related to poor outcome and low survival rates after LT [13]. Therefore, despite that the detection of MVI becomes crucial in patient management, it represents at the same time one of the hardest elements to detect with conventional imaging studies, as they may detect MVI only indirectly based on capsule disruption, irregular tumor margins, peritumoral enhancement, and so on [14]. In this context, nuclear medicine radically changed the decisional algorithm of almost any neoplasm as it allows an *in vivo* assessment of tumor metabolism [15]. In fact, with respect to HCC, despite having a lower sensitivity compared to other types of solid tumors, ^18^F-fluorodeoxyglucose positron emission tomography with computed tomography (^18^F-FDG PET/CT) is capable of reflecting glucose tumor metabolism, and it is used in clinical practice as a useful diagnostic tool for staging or restaging, prognostic stratification, and follow-up purposes [16]. Unfortunately, ^18^F-FDG PET/CT has two main drawbacks related to the highly variable tracer uptake in HCC and the relatively high physiological liver uptake, which limits its use for primary diagnosis [17]. In this paper, we analyze the possible role of ^18^F-FDG uptake as a preoperative imaging biomarker capable of predicting MVI in patients with HCC, thus selecting optimal candidates for LT.

## 2. Tumor Markers as Predictors of MVI

For patients with HCCs outside MC, long-term survival is still achievable as long as MVI is absent or neoadjvuant LRTT is possible. In fact, Mazzaferro et al. explored the survival of patients with tumors outside MC to assess whether the criteria could be expanded and to derive a prognostic model based on specific tumor characteristics [6]. Interestingly, based on the data of 1556 patients from 36 centers who underwent LT for HCC, they reported that, in the group exceeding MC, patients within the up-to-seven criteria (UTS: seven as the sum of the largest nodule diameter in cm and the number of nodules) and without MVI, showed a 5-year survival rate similar to that of patients within MC. This result points out the importance to detect and quantify MVI at a preoperative stage. However, the first line of investigation to expand MC and to increase the preoperative comprehension of HCC tumor aggressiveness lies in biohumoral markers, the most important of which, AFP, has shown to be important not only in the diagnosis of HCC and in the definition of biological behavior but also in predicting outcomes after LT. In particular, Fujiki et al. demonstrated better biological behavior and recurrence-free survival (RFS) rates in patients with AFP levels below 200 *µ*g/L, reaching up to 90% RFS at a 5-year period compared to the increased risk of MVI and 40% 5-year RFS rate with AFP values higher than 800 *µ*g/L [18].

Based on this evidence, Toso et al. investigated a population of 6478 patients and tested several variables to define which could predict patient survival [19]. Their result showed that only total tumor volume (TTV) and AFP level could provide a statistically significant relationship. Therefore, they proposed a composite score with patients with TTV lower than 115 cm^3^ and AFP lower than 400 ng/mL being inside criteria versus patients with higher values. The combined TTV/AFP score efficiently predicted post-LT survival (HR = 2, 95% CI = 1.7–2.4, *P* ≤ 0.001) with a 3-year survival up to 65%, whereas patients outside these criteria had a survival below 50%. However, it is important to highlight that a considerable percentage of HCC patients is AFP-negative and also that a static and single assessment of serum AFP levels cannot express dynamic changes in tumor behavior. In this sense, time-spaced evaluations are necessary to define properly the biological status of the tumor, whether stationary or progressing.

Apart from AFP, another important biomarker useful for the early diagnosis of HCC is protein induced by absence of vitamin K or antagonist II (PIVKA-II), also known as des-*γ*-carboxyprothrombin (DCP). Its prognostic role is linked to its association with tumor growth rates, more in particular with elevated cellular proliferation, infiltrative patterns, and vascular invasion. In addition, it appears to be more sensitive and specific than AFP and also demonstrated to be predictive of outcomes regardless of treatment. In this sense, Fujiki et al. proposed the Kyoto criteria and evaluated the role of simultaneous testing of both markers, DCP, and AFP, in selection criteria for LT in addition to tumor size and number [18, 20]. A ROC curve-based analysis showed that the abilities of preoperative DCP and AFP to predict HCC recurrence after LT were superior to tumor size or number even if the difference was not statistically significant. Their analysis showed optimal cutoff values to predict recurrence at 400 mAU/mL for DCP, 800 ng/mL for AFP, 5 cm for tumor size, and 10 for tumor number, thus concluding that preoperative DCP assessment offers additional information on tumor biological behavior and could be useful in the effort to expand selection criteria for LT in patients with HCC.

## 3. Vascular Invasion as a Predictor for Liver Transplant

Apart from number and size of lesions and biomarker levels, which already have been associated with poorer prognosis, overall survival and recurrence rates after LT, are affected also by the presence of vascular invasion. In particular, it is already well known how macrovascular invasion (macroVI) is associated with reduced outcome after LT in case of HCC; the role of microvascular invasion instead, is yet to be clarified, since it suggests a poor prognosis but it is often hard to detect before transplant [21]. Firstly, it is crucial to underline more in detail the differences between macroVI and MVI [22]. Simply, the former is defined by the presence of gross tumor thrombi and can be detected by conventional medical imaging. On the contrary, the latter can only be seen directly via microscopy, and its definition is more challenging since there are no universally accepted criteria with each study group that proposed its own parameters. In particular, they tried to provide a quantitative assessment of MVI in order to obtain a significant predictive value for recurrence and LT success [23].

Sumie et al. [24] proposed to diagnose MVI once tumor cells were found invading into portal or hepatic venous systems, and they further classified MVI into three degrees according to the number of MVI, that is, no MVI, mild MVI (1–5 MVI), and severe MVI (>5 MVI). However, this method failed to take into account two aspects that proved to be critical, namely, the distance between invaded vessels and edge of the tumor, and whether the tumor had invaded other vascular systems, bile ducts, and lymphatic vessels [25]. On the contrary, Roayaie et al. reviewed 131 patients that underwent resection for HCC and subsequent histological analysis [26]. They showed that invasion of a vessel with a muscular wall, invasion of a vessel ≥1 cm from the tumor capsule, and invasion of more than five vessels were significantly associated with recurrence. Based on the presence/absence of these two microscopic features of MVI, they elaborated a risk score assigning one point for invasion of a vessel with a muscular wall and one point for invasion of a vessel ≥1 cm from the tumor capsule, which was found to significantly and independently correlate with risk of recurrence and death. Then, they divided their patients with vascular invasion into five groups: group A, no MVI; B1, presence of MVI alone; B2, MVI with one aggressive feature; B3, MVI with two features; and C, macrovascular invasion. Instead in 2015, the Chinese Society of Pathology proposed a different definition of MVI based on the number of cells that can be found in the endothelial vascular lumen under microscopy that need to be “a nest” of cancer cell, namely, higher than 50. Moreover, they took into account the distance factor which defined three additional subgrades M0, no MVI; M1 (the low-risk group), ≤5 MVI in adjacent liver tissue ≤1 cm away from the tumor; M2 (the high-risk group), >5 MVI or MVI in liver tissue >1 cm away from the tumor [27].

With respect to MVI predictive value, Esnaola et al. reported one of the first evidences concerning its role in 2002, studying a cohort of 245 patients who underwent resection for HCC and were candidates for LT [28]. They reported a strong correlation between tumor size (greater than 4 cm), tumor grade (poorly differentiated), and MVI, with an odds ratio (OR) of 3.0 and 6.3, respectively [28]. Therefore, they concluded that a tumor biopsy with pathologic grading at a pretransplant stage could significantly improve the selection of patients with HCC for LT. In 2007, Shah et al. evaluated 155 patients with confirmed HCC after LT that satisfied Milan criteria in a 13-year period and defined MVI based on pathologic analysis, intended as microscopic vascular invasion of small vessels within liver parenchyma [29]. Their analysis showed that MVI was significantly associated with both number and size of tumors and that 68% of all patients who developed HCC recurrence were positive to MVI. More recently, in 2016, Jakhete et al. evaluated the possible role of MVI in HCC and LT [30]. In particular, they studied 107 patients that underwent liver transplant in a 13-year period at Johns Hopkins Medical Center, reporting 22% of patients with MVI. The rate of poor differentiation and alphafetoprotein level above 1000 ng/mL were more common in this group, but based on univariate and multivariate analyses, these factors did not reach statistical significance in predicting MVI on pathology. Despite the diagnostic value of routine pathological assessment of MVI, the risk of tumor seeding represents a significant drawback in pre-LT setting; therefore, it is possible to understand how MVI still represents a field of utmost interest since its prediction can be a crucial aspect in a better definition of patients for LT.

## 4. Diagnostic Imaging and Vascular Invasion

In this context, diagnostic imaging and tumor markers may play an important role and become the main tools to define MVI. In particular, a possible role could be found for computed tomography (CT), magnetic resonance imaging (MRI), and positron emission tomography (PET) [31].

### 4.1. Contrast-Enhanced CT

Contrast-enhanced CT examinations have a high sensitivity and allow the detection of hypervascular HCCs as small as 3 mm [32]. Triple-phase CT scan of the liver during contrast injection include an arterial phase shortly after injection, a portal phase, and a delayed phase [33]. It can visualize tumors that are isoattenuated on the arterial and portal phases, increasing the sensitivity. The arterial phase will highlight arterial lesions whereas the portal phase will show lesions that primarily enhance from the portal vein [34]. The portal phase shows the liver during higher parenchymal enhancement and allows depiction of most lesions with a greater lesion-to-liver ratio. The delayed phase of helical CT is performed 5 to 10 minutes after contrast and has been reported to show higher liver HCC contrast than does the portal-venous phase, thus improving the rate of detection of well-differentiated hypovascular HCCs. Chou et al. prospectively enrolled 102 patients with preoperative multiphasic CT findings of solitary HCC [35]. Numerous morphologic parameters were assessed, but the main correlation they evaluated was between histopathologic findings of MVI and tumor margins on preoperative CT images. Their multivariate analysis results showed that, among all parameters, only nonsmooth margins correlated with the presence of MVI in HCC (*p* < 0.001); in particular, out of 60 pathologic examination that revealed MVI, 40 of them exhibited focal nonsmooth margins, with a similar pattern of localization in 36 cases. The current standard method to assess response of unresectable primary and metastatic HCC is by contrast-enhanced CT using response evaluation criteria in solid tumors (RECIST) criteria but it is recognized that this is a relatively insensitive and nonspecific method [36].

Patients with HCC beyond Milan criteria can be downstaged by LRTT. Locoregional therapy serves two objectives for HCC candidates of LT. Firstly, in patients within MC, it can serve as a bridge to transplant to prevent dropout from a waiting list due to tumor progression. Secondly, in patients initially beyond MC, it can serve to reduce tumor size to downstage patients and fulfill MC. Neoadjuvant therapies such as transarterial chemoembolization, radiofrequency ablation, percutaneous ethanol injection, and transarterial radioembolization are used for these purposes. The treatment response has been evaluated radiologically by the modified RECIST [37].

### 4.2. CE-MRI and Diffusion-Weighted Imaging

The sensitivity of MRI to detect HCC is similar to helical CT; however, it is more sensitive and specific in differentiating regenerative nodules from HCC in the cirrhotic liver [38].

Microvascular invasion (MVI) is a powerful, validated, independent predictor of early recurrence and poor overall survival after the surgical treatment of HCC. Other authors have reported that the “typical dynamical pattern,” “nonsmooth tumor margin,” and “apparent diffusion coefficient (ADC) values” are closely associated with MVI. Diffusion is a physical process that results from the thermally driven, random motion of water molecules. DWI and ADC values provide information related to the tissue cellularity and integrity of cellular membranes, as well as microcapillary perfusion, by reflecting the molecular diffusion of water and perfusion [39]. In 2011, Chandarana et al. tried to correlate the presence of MVI at histopathologic examination in patients with HCC that are undergoing LT, with clinic-pathologic and MRI parameters. In particular, they studied 60 patients who underwent a pretransplant MRI (within 90 days before the actual liver transplant) and several morphologic parameters were taken into consideration including number, size, T1 and T2 signal intensities, margins, distance to closest vessel, and so on [40]. However, their results demonstrated that none of the parameters were predictive of MVI but tumor multifocality, in particular the presence of three or more lesions on MRI, had a specificity of 88.2% in predicting MVI, with an OR of 2.43 (*p*=0.013). In 2018, C. Yang et al. explored the potential use of MRI for predicting the MVI of multiple HCC lesions. 51 patients with two HCC lesions in different hepatic segments lesions were included in their study and was compared the relative diffusion-weighted imaging (DWI) signal intensity (SI) and the relative ADC values of the two lesions to predict MVI in bifocal HCC patients [41]. The conclusion they came to is that, in patients with two HCC lesions, highly similar ADC values for the two HCC lesions may be a preoperative predictor of MVI. Using diffusion-weighted imaging (DWI), Xu et al. showed that lower ADC values (<1.227 × 10^−3^ mm^2^/s) on DWI with *b*-value of 0.500 s/mm^2^ can be a useful preoperative predictor of MVI for small HCCs, providing a sensitivity of 66.7% and a specificity of 78.6% [42]. Another method to assess the presence of MVI is using MRI with hepatospecific contrast medium. Gadoxetic acid (Gd-EOB-DTPA, Primovist; Bayer Schering Pharma AG, Berlin, Germany) is a relatively new, safe, and well tolerated liver-specific contrast agent for magnetic resonance imaging (MRI) of the liver that allows for the acquisition of both dynamic and hepatobiliary phase images [43]. Recently, it has been suggested that the peritumoral decreased uptake area (PDUA) in the hepatobiliary phase of Gd-EOB-DTPA-enhanced MRI could be observed in cases of impaired hepatocyte function induced by decreased portal flow. PDUA was defined as the presence of a faint and hypointense area around the tumor in the hepatobiliary phase of Gd-EOB-DTPA-enhanced MRI. The three suggested types of PDUA in the hepatobiliary phase of Gd-EOB-DTPA-enhanced MRI are the following: wedge-shaped, irregular belt-shaped, and linear PDUA [44]. Previous reports have suggested that PDUA in the hepatobiliary phase of MRI may be associated with vascular invasion in HCC. However, whether PDUA in the hepatobiliary phase of Gd-EOB-DTPA-enhanced MRI has a clinically significant effect on the incidence of tumor recurrence following resection remains unclear. Shin et al. in 2017 conducted a study to investigate correlations between MVI and PDUA in the hepatobiliary phase of Gd-EOB-DTPA-enhanced MRI and to elucidate the predictability of PDUA for tumor recurrence after resection in patients with a single HCC 5 cm in diameter without macroVI [45]. They have shown that PDUA in the hepatobiliary phase of Gd-EOBDTPA-enhanced MRI could be a useful preoperative predictor of MVI and an independent prognostic factor after surgical resection in patients with a single HCC 5 cm in diameter without macroVI [46]. Kim et al. arrived at the same conclusions, showing that peritumoral hypointensity seen on hepatobiliary phase images of EOB-MRI can be useful for predicting MVI of HCC preoperatively. The presence of peritumoral hypointensity showed high specificity (93.2%) and a PPV of 88.5% [47]. This relationship may be explained by decreased uptake of gadoxetate disodium by hepatocytes due to obstruction of minute portal branches by tumor thrombi, resulting in hemodynamic changes. Lower ADC values on DWI and a hypointense area around the tumor in the hepatobiliary phase of contrast-enhanced MRI are considered as useful preoperative predictor of MVI. In conclusion, preoperative identification of MVI is important when determining the best candidates for surgical resection and/or liver transplantation and predicting postoperative outcome.

### 4.3. CEUS

The sensitivity of baseline US in the detection of HCC is limited ranging from 40% to 70%, for lesions below 2 cm in diameter. In particular, to be detected, the nodule has to present different ultrasonic properties from the surrounding parenchyma, hence either liver atrophy or the interposition of bowel gases could reduce the accessibility of liver tissue, as well as fibrosis, steatohepatitis, and micro/macronodules could limit the study of deeper segments [48]. In this sense, contrast-enhanced ultrasound is a relatively recent imaging technique that allows real-time recording and evaluation of the wash-in and wash-out phases of the ultrasound contrast agent over several minutes, which is represented by either a bolus injection of microbubbles or an intravenous infusion with the disruption-replenishment technique, thus allowing a quantitative assessment thanks to time intensity curves. With respect to liver examination, the European Federation of Societies for Ultrasound in Medicine and Biology (EFSUMB) in its own guidelines recommends a CEUS study to characterize any lesion or suspect lesion detected at baseline US in the setting of liver cirrhosis. In particular, thanks to low mechanical index (MI) imaging technique, it allows continuous detection of the microbubbles during all three different vascular phases that define liver transit, namely, arterial, portal-venous, and late-sinusoidal phase [49]. This is of utmost importance as it grants the assessment of vascular architecture in the early wash-in phase and the contrast enhancement of the lesion compared to the adjacent normal tissue in the period between wash-in and wash-out. Therefore, thanks to the abovementioned diagnostic features, CEUS can characterize focal liver lesions, differentiating either malignant or benign. The former shows a hypoenhancement in the portal-venous phase with respect to the surrounding healthy liver parenchyma, whereas benign lesions tend to have an iso- or hyperenhancement in the sinusoidal-hepatospecific phase compared to the surrounding tissues.

In fact, with respect to HCC, CEUS provides a tool to show arterial neoangiogenesis, which has been observed in 91–96% of HCC lesions, as it usually shows a strong intratumoral enhancement in the first 25–35 seconds after contrast media injection (i.e. arterial phase) followed by rapid wash-out, with an isoechoic or hypoechoic pattern in both the portal-venous and delayed phases [50]. Approximately 70% of HCCs will become hypoechoic during late-phase imaging, depending upon cellular differentiation, with well-differentiated lesions that tend to be instead isoechoic. Consequently, CEUS kinetics is of critical importance as it allows characterization of other focal liver lesions, such as regenerating and dysplastic nodules and hemangiomas. For instance, regenerating nodules do not exhibit any strong enhancement during the arterial phase, but they enhance with the surrounding nontumoral parenchyma and they typically disappear during portal and delayed phases. Hemangiomas instead, typically do not show any vascularity at color Doppler US but they exhibit a very specific enhancement pattern with peripheral globular enhancement during the arterial phase. In terms of sensitivity, in a recent study, CEUS reached very high values in the detection of arterial hypervascularity and also related to dimensions up to 97% in lesions >3 cm, 92% in lesions ranging 2-3 cm, 87% in lesions ranging 1-2 cm, and 67% in lesions <1 cm. Therefore, a CEUS study could be suggested without hesitation in all suspected lesions ≥1 cm in diameter detected at baseline US in patients with cirrhosis or chronic hepatitis in follow-up [51].

In addition, CEUS is useful in case of small nodules (below 2 cm) that can be particularly challenging from a diagnostic point of view, requiring most of the times a multimodality-based approach. In this sense, CEUS could offer unique advantages over CT and/or MRI such as a sensitive depiction of arterial hypervascularity of HCC, an easier differentiation of non-HCC and HCC lesions thanks to a different washout pattern, rapid and very late respectively. With respect to MVI instead, CEUS might not be the method of choice because MVI apart from the adjacent peritumoral tissues can be located also in distant peritumoral vessels, at more than 1 cm of distance, thus underlining the importance of a multimodal approach, with global techniques such as CT or MRI that analyze the liver in its entirety [52].

### 4.4. Superb Microvascular Imaging (SMI)

A novel vascular imaging model based on the Doppler technique is very useful in the real-time visualization of microvascular flow and slower blood flows in general, thanks to an advanced clutter suppression algorithm, high frame rates, and high-resolution images [53]. In other words, with SMI, it is possible to visualize small vessels and their branches that were only visible via CEUS [54]. In a recent paper, Ruigang et al compared SMI with conventional ultrasound to evaluate thyroid nodules, which are known to be characterized by microvascular flow. Their results showed that SMI revealed more small branches of microvasculature with respect to conventional color-doppler imaging and power-doppler imaging and was capable of defining the distribution both inside the nodules and in the adjacent thyroid parenchyma in better detail. In an analogous way, it could be interesting the application of SMI to HCC nodules in order to better characterize their microvascular distribution and flow [55].

## 5. The Role of ^18^F-FDG-PET

If a morphology-based approach seems to be an important starting point for the detection of MVI, a functional/metabolic assessment by using nuclear medicine techniques may represent the natural development. In this sense, the state-of-art imaging systems are hybrid machines such as PET/CT and PET/RM, which combine the best morphological and metabolic tumor characterization in one exam [56, 57]. Therefore, the focus of the literature is now pointed at the definition of the relationship between grade of differentiation and FDG uptake; in particular, it is generally assumed that the false-negative rate is high in well-differentiated HCCs, whereas poorly differentiated carcinomas present avid and focal increase of glucose metabolism. Wu et al. showed sensitivity for ^18^F-FDG in well differentiated and moderately differentiated HCCs is as low as 35%, but 83.3% in poorly differentiated HCC. The high level of FDG-6-phosphatase activity in well-differentiated HCCs appears to be responsible for the high false-negative rate, as FDG-6-phosphate is not trapped in the cell [58].

From a pathophysiological point of view, it is very interesting to understand the mechanism behind ^18^F-FDG uptake at the tumor level, compared to normal liver tissue. One of the biochemical characteristics of malignant cells is an enhanced rate of glucose metabolism due to increased number of these cell surface glucose-transporter proteins (such as Glut-1 and Glut-3) and increased intracellular enzyme levels of hexokinase and phosphofructokinase which promote glycolysis. FDG is phosphorylated to FDG-6-phosphate, which, unlike glucose-6-phosphate, cannot be metabolized further, and remains trapped in the cell. In normal liver tissue, activity of the enzyme glucose-6-phosphatase, which converts FDG-6-P to FDG is high, whereas it is very low in liver metastasis, resulting in an increased FDG uptake pattern on the PET scan [59]. In contrast, the enzyme activity varies considerably among different types of HCC: well-differentiated HCC nodules exhibit an enzyme activity that is comparable to normal liver tissue [17]. Therefore, low-grade tumors tend to have a similar FDG uptake pattern than the surrounding normal tissue, finally leading to a low standard uptake value (SUV). On the contrary, increased FDG uptake may be visualized in poorly differentiated HCC. Consequently, several studies have reported only a modest (below 50%) sensitivity of 18F-FDG-PET for diagnosing HCC [60].

Therefore, the other important aspect of ^18^F-FDG PET/CT in HCC patients is related to the technical issue of normal liver ROI positioning for SUV assessment. Different approaches have been tried each with its own characteristics. Hyun et al. obtained normal liver SUVmean as the average of three VOIs (two in the right lobe and one in the left lobe) of 1 cm diameter located where HCC was not detected and avoiding at the same time beam-hardening artifacts, focal changes of fatty liver, major vessels, bile ducts, and liver surface margins [61]. Boussouar et al. collected for each HCC nodule that showed increased FDG uptake several parameters including apart from the location of the tumor, maximum SUV of the tumor (SUVT), and maximum SUV of the normal liver parenchyma (SUVL) [62]. The former was calculated taking into account five contiguous slices around each focus of tumor uptake for which ROIs were drawn; the latter instead was extrapolated by ROI drawn at midheight, making sure that VOI margins avoided sites of disease thus being limited to areas of physiologic uptake. From these two values, they obtained a tumour-to-liver ratio (SUVT/L) for each lesion. Finally, Kornberg et al. compared any significantly enhanced 18F-FDG uptake focus to normal adjacent liver tissue and in a direct approach a tumor to nontumor SUVmax higher than 1 defined a positivity status for HCC of PET scan [16].

In addition to solving technical issues related to ^18^F-FDG PET/CT for HCC patients, the authors' efforts were directed also in the definition of ^18^F-FDG PET/CT prognostic value, in particular in patients undergoing LT. A recent literature revision analyzed sixteen studies for a total number of 1157 patients; the results consistently reported a significantly shorter recurrence-free survival in the group of patients with ^18^F-FDG-positive HCC with respect to ^18^F-FDG-negative [63]. Therefore, the author concluded that, in the included studies, ^18^F-FDG PET/CT showed a high prognostic value. These studies, although mainly retrospective, showed promising results, with negative preoperative PET that could predict up to a three-year recurrence-free survival post-LT, even in patients not responding completely to MC; high positive and negative predictive values have also been reported, 87.5% and 88.5%, respectively, for ^18^F-FDG uptake on PET scans. In another retrospective study [64], Detry et al. analyzed 27 patients with HCC who underwent a pre-LT ^18^F-FDG PET/CT scan and found a statistically significant difference when comparing recurrence-free survivals with respect to ^18^F-FDG uptake. In particular, via a ROC curve analysis, they identified an optimal semiquantitative cutoff of tumor/liver activity ratio (RSUVmax) at 1.15, a value that acts as strong prognostic factor for both recurrence and death events in patients with HCC treated by LT, even in patients outside MC.

Ahn et al. instead investigated the possible role of gadotexic-acid-enhanced MRI and ^18^F-FDG PET/CT in the prediction of MVI in 51 patients with HCC prior to LT. With respect to MRI findings, among all parameters considered preliminarily the only ones that showed a statistically significant association with MVI were hypointensity on T1WI, peritumoral enhancement, inhomogeneity in the arterial phase, in the delayed phase, or in the HBP, and the large tumor size [65]. With respect to ^18^F-FDG PET/CT findings, as already highlighted by other studies, PET-positive HCCs were significantly more frequent and with higher SUVmax and RSUVmax values in the MVI-positive group than in the MVI-negative group. In particular, using the same aforementioned ROC curve analysis, they selected a critical cutoff value for RSUVmax at 1.2 due to its high specificity, namely, 86.6%. In this sense, in the MVI-positive group, 71.4% of lesions showed a RSUVmax equal to or greater than 1.2. Multivariate analysis of both MRI and ^18^F-FDG PET/CT revealed that in the prediction of MVI peritumoral enhancement and RSUVmax of 1.2 or greater were the two statistically significant parameters to take in consideration, with an OR of 10.648 and 14.218, respectively. Unfortunately, neither modality provided sufficient sensitivity or specificity, with gadotexic-acid-enhanced MRI that reached a sensitivity of 35.7% and a specificity of 93.3% and RSUVmax ≥ 1.2 at ^18^F-FDG PET/CT that showed a sensitivity of 64.3% and a specificity of 86.7%. However, when combining both information, it was possible to predict MVI of HCCs with high sensitivity and specificity values, 78.6% and 80%, respectively.

## 6. The Importance of Downstaging: When LT Is Feasible?

Thanks to the success of LT for early stage HCC, modest expansion beyond MC has been proposed to increase eligibility for LT. In this sense, tumor downstaging may facilitate LT for patients with HCC outside MC; however, there is no optimal protocol yet and downstaging outcomes are poorly defined [66]. Tumor downstaging can be defined as tumor size reduction using locoregional therapies (LRT), including transarterial chemoembolization (TACE) or radiofrequency ablation (RFA), which are used as a bridge in order to meet acceptable criteria for LT. Therefore, it could theoretically help in the selection of a subgroup of patients with HCC that initially exceeds transplant criteria but that will probably do well after LT. As recent studies confirmed, another crucial factor is represented by tumor biology, which can be summed up in two prognostic elements, poor differentiation, and MVI that are associated with recurrence in patients within and beyond various transplant criteria. Conversely, the absence of these risk factors may justify LT also in patients with larger HCC [67].

The first line of LRT employed in the majority of published papers in patients undergoing tumor downstaging has been TACE, due to the presence of large lesions and/or multifocal disease, with multiple TACE sessions that are usually required to target all active disease foci. In case of nonresponders to TACE, LRT based on local ablative techniques may be considered if tumor location is feasible for treatment and if there are no more than three foci of residual/recurrent disease. In particular, percutaneous radiofrequency ablation (RFA) is the most commonly used technique that achieves best results in lesions <3 cm in maximal diameter [68].

Since tumor downstaging should be useful to select tumors with favorable biology and better prognosis, and HCC recurrence rate and the incidence of MVI and/or poorly differentiated tumor grade (unfavorable histologic characteristics) should be comparable to those of patients meeting LT criteria without downstaging. In this sense, Yao et al. [68] reported the incidence of microvascular or macrovascular invasion in the explant, which was 3.2% in the downstaged group versus 6% in the MC group and poorly differentiated tumor grade that was not found in downstaged patients versus 8.5% incidence in the MC group. Moreover, several studies evaluated this downstaging scenario in patients exceeding the MC and found 5-year overall survival rates not significantly lower than in patients within MC. In particular, Yao et al. studied 118 patients with tumor stage exceeding T2 criteria (one lesion between 2 cm and 5 cm or two to three lesions <3 cm) that underwent downstaging to fulfill MC. Their downstaging protocol included patients without any kind of vascular invasion at imaging exams and meeting one of the following criteria: single lesion ≤8 cm; two or three lesions each lower than 5 cm (sum ≤ 8 cm); and four or five lesions each lower than 3 cm (sum ≤ 8 cm). Their results showed that in 65% of patients, namely, in 77 of 118 patients enrolled into the protocol, the downstaging approach was successful, and 64 patients (54%) subsequently received LT; the 5-year post-LT survival was 78% with an 8% recurrence rate, after a median follow-up of 3.8 years posttransplant. Therefore, by comparing the results to a control group of 488 patients with HCC listed for LT that met MC from the beginning, over the same period, they concluded that there was no significant difference in terms of post-LT survival and recurrence-free probabilities [69].

## 7. Discussion and Conclusions

Hepatocellular carcinoma (HCC) represents the fifth most common cancer and the second leading cause of cancer-related deaths worldwide. Being the curative options limited to surgical resection and liver transplantation (LT), the diagnostic approach to this type of tumor and the selection of patients for therapy become of utmost importance. Mazzaferro et al. published in 1996 the first version the Milan criteria, which led to an improvement in both overall and disease-free survivals, despite being recently criticized because they appear over conservative hence limiting the number of patients that can benefit from a possible curative treatment.

In order to expand MC, the first line of investigation is represented by biohumoral markers, namely, AFP and DCP, which have shown to be important not only in the diagnostic stage but also in biological behavior definition and outcome prediction after LT. The Kyoto criteria for patient selection in LT proposed by Fujiki et al. defined the role of simultaneous testing of both DCP and AFP in addition to tumor size and number. A ROC curve-based analysis showed optimal cutoff values to predict recurrence at 400 mAU/mL for DCP and 800 ng/mL for AFP, thus concluding that preoperative biomarkers assessment might be useful in the effort to expand selection criteria for LT in patients with HCC.

The next step in the goal of expanding MC for LT lies in the definition of vascular invasion, either macroVI or MVI. While macroVI is detectable by conventional imaging techniques, MVI presence is harder to detect with certainty also because there are no universally accepted criteria. To date, the gold standard for MVI detection remains the pathological assessment, despite pretransplant biopsy procedures have shown limited accuracy and high risk of tumor cell seeding. In fact, it appears as nonsense in the effort of proper HCC patient management, since several studies suggest that preoperative liver fine-needle aspiration biopsy (FNAB) is also related to significantly higher recurrences rates, both overall and extrahepatic, when comparing HCC patients with and without FNAB. Nevertheless, the Chinese Society of Pathology recently recommended a sampling process including sites, distances, and volumes for tissue sampling trying to overcome or at least to limit the seeding problem and in order to make more accurate diagnoses based on histological examinations, including the presence of MVI. In particular, they recommend a 7-point baseline sample collection protocol with at least four tissue specimens sampled at tumor margins at 12, 3, 6, and 9o'clock, which is critical to evaluate objectively the biological behavior of the tumor since it is an area rich in highly invasive cells. Their recommendations include at least one specimen sampled at the core of the tumor, in the intratumoral zone, at least one that represents adjacent peritumoral liver tissues within 1 cm to the tumor, and at least one that represents distant peritumoral liver tissues 1 cm away from tumor.

In this context, diagnostic imaging already plays an important role with conventional techniques such as CT and MRI, further improved by contrast-enhancement features (CECT) and diffusion-weighted imaging (DWI), respectively, or even CEUS and SMI. However, thanks to the additional data provided by nuclear medicine that it could become the main tool to define MVI. In particular, ^18^F-FDG PET/CT, taking advantage of the intrinsic capability to assess in a semiquantitative way the increased glucose metabolism, already proved its value in several retrospective studies in which a significantly shorter recurrence-free survival was found in the group of patients with ^18^F-FDG-positive HCC with respect to ^18^F-FDG-negative. In this sense, Kornberg and colleagues systematically reviewed most important works on the role of PET in liver transplant patients with HCC and were able to reach a decisional algorithm that uses ^18^F-FDG PET/CT as a pivotal criterion to point patients towards neoadjuvant LRTT and/or LT, for both patients within and outside morphometric standard criteria, that is, MC. Moreover, based on their findings, the same group also suggested their own novel expanded criteria to select patient for LT, which include within or beyond MC + AFP level <115 ng/ml + negative PET status, reporting a 5-year recurrence-free survival rates of 75% and 44% in patients meeting and exceeding them, respectively (*p*=0.003). The aforementioned promising results that were confirmed even in patients nonresponding completely to MC could be the first step towards an organized extension of MC with a preoperative PET/CT scan that could be added to the decisional algorithm in the effort to improve the early detection of MVI and therefore selection of LT.
